# Thymoglobulin induction in kidney transplantation: real-world cost-effectiveness in Brazilʼs public healthcare system

**DOI:** 10.1590/2175-8239-JBN-2025-E005en

**Published:** 2025-03-10

**Authors:** Roberto Ceratti Manfro

**Affiliations:** 1 Hospital de Clínicas de Porto Alegre, Divisões de Nefrologia e Transplante, Porto Alegre, RS, Brazil.; 2 Universidade Federal do Rio Grande do Sul, Faculdade de Medicina, Porto Alegre, RS, Brazil.

In this issue of BJN, Bessa et al.^1^ present their article entitled “Real-world setting cost-effectiveness analysis of thymoglobulin versus no induction therapy in adult kidney transplant recipients with low risk for graft loss”. The study uses real-world data to evaluate the cost-effectiveness of a proposed induction therapy strategy with a single dose of 3 mg/kg r-ATG (rabbit anti-thymocyte globulin, Thymoglobulin®), a polyclonal antibody preparation that primarily targets T lymphocytes. This immunosuppression strategy was proposed, developed, and validated by the authors in their extensive kidney transplant program^2,3,4^ and has been shown to be equivalent in preventing acute rejection and ensuring equivalent graft survival, with a lower incidence of bacterial infections, in comparison to a full dose regimen, in another Brazilian cohort study^5^. The study was conducted in first-time kidney transplant recipients with low immunological risk who had either living or deceased donors. The outcomes acute rejection, cytomegalovirus infection, graft loss, retransplantation, and death were evaluated using a well-balanced comparison group of patients who did not receive induction therapy. The authors concluded that the proposed therapy was cost-effective within the funding framework of the Brazilian national public healthcare system.

In the study, the authors used the incremental cost-effectiveness ratio (ICER), a key measure in health economics, to compare the cost-effectiveness of an intervention with an alternative by evaluating the additional costs and health benefits. The effectiveness of each intervention refers to its health outcomes, which can be measured, for example, in terms of life years gained, as in the present study. ICER is often compared to a willingness-to-pay (WTP) threshold to determine whether an intervention is cost-effective. If the ICER is below the WTP threshold, the intervention is considered cost-effective; if it exceeds the threshold, it may not be considered cost-effective. However, the methodology has limitations. For instance, ICER does not account for all societal benefits, the WTP threshold can be subjective and varies across countries or decision-makers, and most importantly, the approach requires accurate data on both costs and health outcomes^6^. Additionally, the study employed a Markov model to simulate treatment outcomes and cost-effectiveness, along with sensitivity analyses to strengthen the robustness of the results.

The main results of the study indicated that, within one year post-transplant, there was a significant reduction in the incidence of acute rejection, with a number needed to treat (NNT) of 2.7 patients receiving r-ATG to prevent one episode of acute rejection. However, this benefit came at a significant financial cost per episode avoided. The interpretation of this NNT should be approached with caution, given the relatively high incidence of acute rejection observed in the r-ATG untreated cohort. This incidence might be unexpected in kidney transplant recipients on a Tacrolimus-based regimen, as seen in contemporary cohorts^7,8^. Another key finding during the first year was a minimal improvement in graft survival, achieved at a low incremental cost. The kidney graft survival outcomes at 4 and 10 years were an additional 0.06 and 0.16 years, respectively, and these improvements were associated with enhanced cost-effectiveness.

The interpretation of the study and its external validity should consider some limitations. The study was retrospective, conducted using a quasi-experimental design with a non-contemporary control group, in a real-world setting. As such, it carries the risk of selection bias due to the lack of randomization and the potential influence of confounding variables on the observed outcomes. Additionally, it is a single-center study involving standard immunological risk recipients of a first kidney transplant, either from living donors or from standard-criteria deceased donors, and using Azathioprine—a drug that is no longer the predominantly employed adjunctive agent in contemporary immunosuppression regimens. Despite these limitations, the authors note that the study reflects characteristics that are representative of the transplant recipient population, their respective donors, the type of procedures, and the financial constraints specific to kidney transplantation practices in Brazil.

The study represents another significant contribution by the authors to the development and implementation of this immunosuppression strategy, offering valuable insights for stakeholders such as healthcare systems, public policy makers, and transplant centers.
